# Performance of noninvasive prenatal testing for twin pregnancies in South China

**DOI:** 10.1007/s10815-023-02881-1

**Published:** 2023-07-22

**Authors:** Dongmei Wang, Haishan Peng, Yixia Wang, Yaping Hou, Fangfang Guo, Juan Zhu, Tingting Hu, Jiexia Yang

**Affiliations:** Guangdong Women and Children Hospital, Guangzhou, 511442 Guangdong China

**Keywords:** Twin pregnancies, Chromosome abnormalities, Noninvasive prenatal testing (NIPT), Expanded noninvasive prenatal testing (NIPT-plus), Specificity, Sensitivity

## Abstract

**Abstract:**

**Objective:**

The purpose of this study was to evaluate the performance of noninvasive prenatal testing (NIPT) for the detection of chromosomal aneuploidies and copy number variations (CNVs) in twin pregnancies.

**Method:**

A cohort of 2010 women with twin pregnancies was recruited. 1331 patients opted for NIPT, and 679 patients opted for expanded NIPT (NIPT-plus). All high-risk patients were advised to undergo invasive prenatal diagnosis. All participants were followed up until 6 months after birth.

**Results:**

Twenty-two cases were predicted to have a high risk of chromosome abnormalities by NIPT, of which 14 pregnant women underwent invasive prenatal diagnosis. The 14 cases included 3 cases of trisomy 21, 1 case of trisomy 18, 1 case of trisomy 7, 2 cases of sex chromosome aneuploidies (SCAs), and 7 cases of CNVs, of which the confirmed cases numbered 2, 1, 0, 1, and 0, respectively. Twenty cases were predicted to have a high risk of chromosome abnormalities by NIPT-plus, of which 16 pregnant women underwent invasive prenatal diagnosis. The 16 cases included 1 case of trisomy 21, 1 case of trisomy 7, 7 cases of SCAs, and 7 cases of CNVs, of which were confirmed in 1, 0, 3, and 2, respectively. No false-negative result was reported during the follow-up period.

**Conclusion:**

The NIPT/NIPT-plus has excellent performance in the detection of chromosome aneuploidies in twin pregnancies. But for CNVs, the effectiveness of NIPT is poor, and the NIPT-plus have a certain detection efficiency. It is worth noting that pre- and post-genetic counseling is especially important, and the chorionicity, mode of conception, clinical indications, and fetal fraction should be considered as influencing factors.

## Background

According to the prevention and treatment report of birth defects in China in 2012, the incidence of birth defects in China is approximately 5.6% [1]. Chromosome abnormalities, including chromosomal aneuploidies and copy number variations (CNVs), are important reasons for birth defects, causing mortality or serious disability [2]. Trisomy 21 (T21), sex chromosome aneuploidies (SCAs), trisomy 18 (T18), and trisomy 13 (T13) are the most common aneuploidies. Moreover, CNVs are important factors affecting human phenotypic variations and diseases [3]. A couple with a normal phenotype may have a 1–2% incidence rate of new abnormal CNVs, which is much higher than the incidence rate of Down syndrome (0.1%) [4]. In children with developmental delays and other birth defects, the detection rate of abnormal CNVs is as high as 3% [5]. Therefore, screening for chromosomal aneuploidies and CNVs during genetic consultations and prenatal diagnoses is of great importance.

Noninvasive prenatal testing (NIPT) based on massively parallel sequencing of cell-free fetal DNA (cff-DNA) has rapidly risen in popularity in clinical practice to screen for fetal chromosomal abnormalities. The target screening syndromes of NIPT were originally T21, T18, and T13, and an expanded version of NIPT was developed (named NIPT-plus) to include SCAs and some CNVs. The NIPT sequencing depth is 0.15×, and the data volume is 3 million reads; the NIPT-plus sequencing depth is 0.4×, and the data volume is 8 million reads. Because NIPT/NIPT-plus screens the whole genome, some rare autosomal trisomies (RATs) and CNVs are identified incidentally outside of the detection range. In these cases, clinicians and pregnant women are informed by a relevant supplementary report. In the last decade, a large number of studies have confirmed the high accuracy of NIPT for screening common chromosome aneuploidies in singleton pregnancy. The overall sensitivity of NIPT was 99.17, 98.24, and 100% for T21, T18, and T13, respectively, and the specificity was 99.95, 99.95, and 99.96% for T21, T18, and T13, respectively [6]. According to the latest statement of the American College of Medical Genetics and Genomics (ACMG), NIPT can replace traditional aneuploid screening in different age groups, but women with twin pregnancies and assisted reproductive technology (ART) patients still need to be cautious when choosing this technology [4].

With the increasing maternal age and the increased use of ART in recent years, the incidence of twin pregnancies has shown an upward trend worldwide [7]. Due to the complexity of twin pregnancies, the incidence of chromosomal abnormalities and miscarriage risk associated with invasive diagnosis are higher than those associated with singleton pregnancy. Moreover, routine serological screening has the disadvantage of a low detection rate [8–10]. Thus, women with twin pregnancies need a reliable and effective noninvasive prenatal testing method.

Recently, NIPT-plus, which has a greater sequencing depth than NIPT, has been more frequently used in clinical screening. ACMG's recent guidelines stated that NIPT-plus should not be introduced to routine clinical practice until performance improvements were demonstrated in clinical settings involving large patient cohorts [4]. In this study, we evaluated the performance of NIPT and NIPT-plus for the detection of chromosomal aneuploidies and CNVs in twin pregnancies and analyzed the results considering chorionicity, mode of conception, clinical indications, and fetal fraction as influencing factors.

## Materials and Methods

### Ethics statement

This study followed The Declaration of Helsinki 1964 and was approved by the Ethics Committee of Guangdong Women and Children Hospital (No. 202201088). Every participant received detailed genetic counseling and signed a written informed consent form.

### Participants

This retrospective study enrolled 2010 patients with twin pregnancies in the Medical Genetics Center, Guangdong Women and Children Hospital, China, from January 2015 to December 2020.

The inclusion criteria were as follows: (1) women with live twin pregnancies; (2) women over 18 years old; (3) women who voluntarily accepted NIPT/NIPT-plus for the detection of fetal chromosome abnormalities; (4) gestational age ≥12 weeks; and (5) no abnormal chromosome phenotype in the mother or father.

The exclusion criteria were as follows: (1) the woman had undergone transplantation, xenogenous blood transfusion, xenogenous DNA-based cell immunotherapy, or stem cell therapy performed; (2) the mother or father was a balanced translocation or inversion carrier; (3) a fetus had an abnormal structure as suggested by ultrasound; (4) we had no information (about the complications during pregnancy, pregnancy outcomes and the health of the newborns until 6 months after birth) from telephone calls or follow-up visits; (5) the mother had any malignant tumor while pregnant; and (6) there were other conditions making the doctor concerned about the accuracy of NIPT/NIPT-plus results.

Pregnant women who were eligible underwent NIPT or NIPT-plus according to their preference. The NIPT/NIPT-plus positive cases with no invasive prenatal diagnosis were not included in the evaluation data of test efficacy.

### Experimental Design

Maternal peripheral blood (10 mL) was collected in a blood tube containing ethylenediaminetetraacetic acid (Yangpu Medical Technology, Zhuhai, China) and immediately stored at 4 °C. Plasma was separated within 8 h following a double-centrifugation protocol [11]. If DNA extraction was not performed immediately, plasma was stored at −20 °C. A magnetic bead nucleic acid extraction kit (Magen, Shanghai, China) was used to extract cff-DNA from 400 μL plasma. All subsequent procedures, including library construction, library concentration quantification, and sequencing, were performed according to the instructions of JingXin Fetal Chromosome Aneuploidy (T21, T18, and T13) Testing Kits (CFDA registration permit No. 0153400300) (CapitalBio Technology, Beijing, China). Sequencing was carried out on the BioelectronSeq 4000 semiconductor sequencing platform (CapitalBio Technology). The difference between NIPT and NIPT-plus was that the loading amount of library DNA of NIPT-plus was 2.5 times that of NIPT. The percentage value of cff-DNA assigned to each chromosome was calculated according to a previously reported bioinformatics statistical method, and the *Z* score was used to evaluate the risk of fetal chromosomal aneuploidies [12]. The aneuploidy state of the target chromosome was classified as follows: a *Z* score of more than 4.00 classified a chromosome as affected, a *Z* score of less than 2.58 classified it as unaffected, and a *Z* score between 2.58 (confidence level = 99%) and 4.00 (confidence level = 99.99%) put it in the “gray area”. The data analysis standard and aneuploidy criteria were based on a previous study [13], which demonstrated that the optimized algorithm in massively parallel sequencing was capable of detecting multiple fetal chromosomal abnormalities in both singleton pregnancy and twin pregnancies. The fetal CNVs were identified by fetal copy number analysis through maternal plasma sequencing (FCAPS) [14].

Pregnant women with high-risk results were advised to undergo invasive prenatal procedures by amniocentesis at 18–26 weeks’ gestational age or umbilical cord blood puncture after 26 weeks’ gestational age. The amniotic fluid sample (20 mL) or umbilical cord blood sample (1 mL) was used for chromosome karyotype analysis and chromosomal microarray analysis (CMA) by using the CytoScan 750K chip (Affymetrix, USA).

Pregnant women with low-risk results were recommended to undergo grade III ultrasound examination at 22–26 weeks’ gestational age, routine prenatal examination, and follow-up until 6 months after birth. If obvious structural abnormalities were found in the fetus, invasive prenatal diagnosis was recommended. The pregnancy results were obtained by telephone follow-up or accessing the hospital’s electronic medical records after delivery. If no abnormality was found during the follow-up period, the NIPT or NIPT-plus result were considered as a true-negative result.

In addition, we divided both the NIPT group and the NIPT-plus group into eight groups according to the test indications: Group A: isolated advanced maternal age (≥35 years, AMA); Group B: high risk of serological screening (T21>1/250, T18>1/350); Group C: intermediate risk of serological screening (1/1000<T21<1/250, 1/1000<T18<1/350); Group D: isolated ultrasound soft-marker abnormality (nuchal translucency (NT) thickening (≥2.5 mm), fetal growth restriction, etc.); Group E: serological screening for single marker value abnormality (β-human chorionic gonadotropin (β-hCG), alpha-fetoprotein (AFP), unconjugated estriol (uE3), etc.); Group F: all forms of ART; Group G: previous adverse outcome of pregnancy or previous pregnancy history of chromosomal abnormalities in fetuses; and Group H: voluntary testing.

Because NIPT/NIPT-plus is an indirect test of the fetus, there is a possibility of false-positive or false-negative results. Therefore, follow-up after testing is crucial for evaluating the performance of NIPT/NIPT-plus. According to the guidelines of the National Health Commission of the People’s Republic of China (http://www.nhc.gov.cn/fys/s3581/201611/0e6fe5bac1664ebda8bc28ad0ed68389.shtml), the follow-up covered complications during pregnancy, pregnancy outcomes, and the health of the newborns until 6 months after birth.

### Statistical analysis

The measurement data are presented as the mean ± standard deviation, and the counting data are presented as the incidence frequency (in percentage). The SPSS statistical software package (version 25.0, regression analysis) was used to process the fetal fraction and gestational age data. The *Z* score was calculated by the data processing software.

## Results

### Demographic characteristics and clinical indications of participants

From January 2015 to December 2020, a total of 2010 patients with twin pregnancies who met the inclusion criteria were enrolled for NIPT/NIPT-plus detection in the Medical Genetics Center of Guangdong Women and Children Hospital.

Table 1 shows the basic demographic characteristics and clinical indications of NIPT and NIPT-plus. Of the 2010 pregnant women, 1331 underwent NIPT and 679 underwent NIPT-plus. In the NIPT group, the average gestational age was 17.04±4.03, 73.70% of pregnancies were dichorionic diamniotic (DCDA), and 54.24% of pregnancies were conceived by ART. In the NIPT-plus group, the average gestational age was 16.21±3.91, 75.41% of pregnancies were DCDA, and 54.64% of pregnancies were conceived by ART.

**Table 1:** Demographic characteristics and clinical indications of participants

		NIPT (1331)	NIPT-plus (679)
Maternal age (year)			
	< 35	973 (73.10%)	499 (73.49%)
	≥ 35	358 (26.9%)	180 (26.51%)
Gestational age (week)			
	12~13	262 (19.68%)	201 (29.60%)
	14~27	1025 (77.01%)	462 (68.04%)
	> 28	44 (3.31%)	16 (2.36%)
Chorionicity			
	MCMA	9 (0.68%)	10 (1.47%)
	MCDA	291 (21.86%)	149 (21.94%)
	DCDA	981 (73.70%)	512 (75.41%)
	Unknown	50 (3.76%)	8 (1.18%)
Mode of conception			
	ART	722 (54.24%)	371 (54.64%)
	SC	609 (45.76%)	308 (45.36%)
Groups of clinical indications			
	A: isolated advanced maternal age (≥35 years, AMA)	22 (1.65%)	4 (0.59%)
	B: high risk of serological screening (T21>1/250, T18>1/350)	134 (10.07%)	38 (5.60%)
	C: intermediate risk of serological screening (1/1000<T21<1/250, 1/1000<T18<1/350)	298 (22.39%)	82 (12.08%)
	D: isolated ultrasound soft-marker abnormality (nuchal translucency (NT) thickening (≥2.5 mm), fetal growth restriction, etc.)	68 (5.11%)	82 (12.07%)
	E: serological screening for single marker value abnormality	33 (2.48%)	21 (3.09%)
	F: all forms of assisted reproduction technology	357 (26.82%)	232 (34.17%)
	G: previous adverse outcomes of pregnancy or previous pregnancy history of chromosomal abnormalities in fetuses	33 (2.48%)	39 (5.74%)
	H: voluntary testing	386 (29.00%)	181 (26.66%)

*MCMA*, monochorionic monoamniotic; *MCDA*, monochorionic diamniotic; *DCDA*, dichorionic diamniotic; *ART*, assisted reproduction technology; *SC*, spontaneously conceived

In addition, the most common clinical indications of NIPT in the 1331 pregnant women were voluntary testing, accounting for 29%, followed by ART (26%) and intermediate-risk serological screening (22%). In NIPT-plus, they were ART (34%), voluntary testing (26%), and intermediate-risk serological screening (12.08%).

### NIPT/NIPT-plus positive cases and follow-up results

A total of 42 pregnant women were at high risk of chromosome abnormalities among the 2,010 samples.

Twenty-two screening-positive cases were predicted by NIPT among the 1331 pregnant women (1.65%). Fourteen patients (63.64%, 14/22) underwent invasive prenatal diagnosis, and one pregnant woman did not have follow-up information. Seven pregnant women had no invasive prenatal diagnosis, one pregnant woman had multiple malformations confirmed by B-ultrasound and underwent an abortion, and one pregnant woman had a miscarriage. Among the other five, during the follow-up until 6 months after birth, three patients showed no abnormalities, and two patients had a child with heart disease. The 14 pregnant women who underwent invasive prenatal diagnosis included three cases of T21, one case of T18, one case of trisomy 7 (T7), two cases of SCAs, and seven cases of CNVs, of which the confirmed cases numbered 2, 1, 0, 1, and 0, respectively (Table 2).

**Table 2:** Information of screening-positive cases in the NIPT group

Case	Age (years)	Gestational weeks (week)	Test indications	Chorionicity	conception	Fetal concentration (%)	BMI	NIPT results	Prenatal diagnostic results	Pregnancy outcome
1	35	18+	C	DCDA	ART	10.88	26	4 dup 20Mb	Normal	Two girls were born healthy
2	27	22+	H	MCDA	SC	5.35	25	T21	Normal	Two girls were born healthy
3	28	13+	H	DCDA	ART	13.99	21	18 del 12Mb	Normal	Two girls were born healthy
4	33	16+	C	DCDA	ART	13.10	20	T21	One of the twins T21	The T21 fetus was reduced, and a girl was born healthy
5	21	29+	H	MCDA	SC	39.35	20	5 del 10Mb	Normal	Two girls were born healthy
6	31	12+	H	DCDA	ART	17.71	19	10 del 10Mb	Normal	Two girls were born healthy
7	32	24	H	DCDA	SC	11.25	21	16 dup 2Mb	Normal	A boy and a girl were born healthy
8	37	16	B	DCDA	SC	7.49	22	14 del 9Mb	Normal	A boy and a girl were born healthy
9	33	12+	H	MCDA	SC	20.91	26	T7	Normal	Two girls were born healthy
10	25	24+	F	DCDA	ART	14.81	23	47,XXY	One of the twins mos47,XXX, 21pss(28)/46, XX, 21pss(22)	A boy and a girl were born healthy
11	31	18+	C	DCDA	SC	19.52	24	45,X	Normal	Two girls were born healthy
12	37	16+	B	DCDA	SC	6.07	27	T21	One of the twins T21	The T21 fetus was reduced, and a girl was born healthy
13	31	19+	H	DCDA	SC	9.04	25	T18	One of the twins T18	The T18 fetus was reduced, and a boy was born healthy
14	29	14+	H	DCDA	SC	8.93	19	20 del 15Mb	Normal	A boy and a girl were born healthy
15	26	17+	C	DCDA	ART	6.34	22	T18	No prenatal diagnosis	multiple malformations confirmed by B-ultrasound and underwent an abortion
16	27	20	C	DCDA	ART	14.88	19	10 dup 30Mb	No prenatal diagnosis	stillbirth
17	32	15+	H	DCDA	ART	4.71	24	8 dup 15Mb	No prenatal diagnosis	A boy and a girl were born healthy
18	37	23+	H	DCDA	SC	12.29	21	T7	No prenatal diagnosis	A boy and a girl were born healthy
19	28	14+	H	DCDA	ART	19.24	20	47, XYY	No prenatal diagnosis	Two boys were born healthy
20	37	18	H	DCDA	SC	19.79	30	T21	No prenatal diagnosis	A boy was born with heart disease and a boy was born healthy
21	34	15+	C	DCDA	SC	13.72	24	T18	No prenatal diagnosis	without follow-up information
22	24	18+	B	unknown	SC	19.74	20	47, XXX	No prenatal diagnosis	A girl was born with heart disease, and a girl was born healthy

*MCMA*, monochorionic monoamniotic; *MCDA*, monochorionic diamniotic; *DCDA*, dichorionic diamniotic; *SC*, spontaneously conceived; *ART*, assisted reproduction technology; *A*, advanced maternal age; *B*, high risk of serological screening; *C*, intermediate risk of serological screening; *D*, isolated ultrasound soft-marker abnormality; *E*, serological screening for single marker value abnormality; *F*, all forms of ART; *G*, previous adverse outcomes of pregnancy or previous pregnancy history of chromosomal abnormalities in fetuses; *H*, voluntary testing

NIPT-plus reported 20 screening-positive cases out of 679 pregnant women (2.95%). Sixteen patients (80%, 16/20) underwent prenatal diagnosis, and one pregnant woman did not have follow-up information. Three pregnant women had no invasive prenatal diagnosis. One of these three, case 20, had one twin with growth restriction, so she chose to reduce this fetus later. The other two chose to continue the pregnancies, and no abnormalities were found during the follow-up period. The 16 pregnant women who underwent prenatal diagnosis, including one case of T21, one case of T7, seven cases of SCAs, and seven cases of CNVs, had 1, 0, 3, and 2 confirmed cases, respectively (Table 3).

**Table 3:** Information of screening-positive cases in the NIPT-plus group

Case	Age (years)	Gestational weeks (week)	Test indications	Chorionic	Conception	Fetal concentration (%)	BMI	NIPT-plus results	Prenatal diagnostic results	Pregnancy outcome
1	40	13+	C	MCDA	SC	32.83	23	47,XXY	Both fetuses were 47,XXY	Terminate Pregnancy
2	48	22	H	MCDA	SC	24.15	24	15 dup 2Mb	Normal	A boy and a girl were born healthy
3	35	18	H	DCDA	ART	14.14	25	12 dup 3Mb	Normal	A boy and a girl were born healthy
4	32	12+	G	DCDA	ART	13.69	28	9 dup 9Mb	Normal	Two boys were born healthy
5	38	13+	D (NT thickening)	DCDA	SC	16.52	19	sex chromosome trisomies	One of the twins 47,XXY	A boy and a girl were born healthy
6	24	13+	E	MCDA	ART	18.21	19	16 dup 3Mb	Both fetuses were arr[hg19] 16p13.11p12.3(15,338,152-18,242,713)x3	Two girls were born healthy
7	23	19+	H	DCDA	SC	8.10	21	45,X	Normal	A boy and a girl were born healthy
8	29	16+	C	DCDA	ART	25.63	20	T7	Normal	A boy and a girl were born healthy
9	26	13+	D (NT thickening)	DCDA	SC	13.42	18	45,X	Normal	Two girls were born healthy
10	39	13+	H	DCDA	ART	23.09	24	5 dup 6Mb	Normal	Two boys were born healthy
11	39	12+	F	DCDA	ART	20.97	20	T21	One of the twins T21	The T21 fetus was reduced and a boy was born healthy
12	32	18	G	DCDA	ART	23.63	28	sex chromosome trisomies	One of the twins 47,XYY	Two boys were born healthy
13	28	16+	H	MCDA	SC	15.04	28	3 del 7Mb	Normal	Two boys were born healthy
14	38	15+	B	DCDA	ART	19.41	23	45,X	Normal	Two girls were born healthy
15	31	20	H	DCDA	SC	5.85	25	45,X	Normal	A boy and a girl were born healthy
16	36	17	F	DCDA	ART	15.40	22	13 del 6Mb	One of the twins 13q21.33q22.2(70,648,847-76,558,847)x1	A girl was stillbirth and a girl was born healthy
17	29	16+	H	DCDA	SC	7.86	27	16 del 2Mb	No prenatal diagnosis	Two girls were born healthy
18	39	15	H	DCDA	ART	5.89	24	45,X/XY	No prenatal diagnosis	Two girls were born healthy
19	29	14	H	DCDA	SC	10.95	20	T7	No prenatal diagnosis	Without follow-up information
20	33	16	H	MCDA	SC	29	18	T20 mos	No prenatal diagnosis	One fetus was reduced (growth retardation) and a boy was born healthy

*MCMA*, monochorionic monoamniotic; *MCDA*, monochorionic diamniotic; *DCDA*, dichorionic diamniotic; *SC*, spontaneously conceived; *ART*, assisted reproduction technology; *A*, advanced maternal age; *B*, high risk of serological screening; *C*, intermediate risk of serological screening; *D*, isolated ultrasound soft-marker abnormality; *E*, serological screening for single marker value abnormality; *F*, all forms of ART; *G*, previous adverse outcomes of pregnancy or previous pregnancy history of chromosomal abnormalities in fetuses; *H*, voluntary testing

A total of 10 cases were confirmed to be true-positive. All of them received prenatal genetic counseling. Two pregnant women with CNVs (1 case of 16 dup2.9Mb, 1 case of 13 del5.9Mb) and 3 pregnant women with SCAs (1 case of 47,XXY, 1 case of 47,XYY, 1 case of 47,XXX) chose to continue the pregnancies, and no visible abnormalities were found by newborn screening during the follow-up period. Four pregnant women with common trisomy abnormalities (3 cases of T21, 1 case of T18) and 1 with SCAs (47,XXY) chose to reduce the affected fetus.

### NIPT/NIPT-plus negative cases and follow-up results

For the NIPT/NIPT-plus negative pregnant women, the invasive prenatal diagnosis was based on their clinical indications and their willingness to undergo the diagnostic procedures. A total of 1968 negative cases in this study were followed-up. There were no prenatal diagnostic results related to chromosomal abnormalities within the scope of this study, and no visible abnormalities were found in the newborn screening in the follow-up period. The success rate of follow-up was 100%.

Since no false-negative result was reported during the 6-month follow-up after birth, the sensitivities and specificities were above 99% in the NIPT group and in the NIPT-plus group, as shown in Table 4.

**Table 4:** Detection efficiency of chromosomal conditions in the NIPT group and the NIPT-plus group

	NIPT						NIPT-plus				
	T21	T18	T7	SCAs		CNVs	T21	T7	SCAs		CNVs
				45,X	47,XXX/47,XXY/47,XYY				45,X	47,XXX/47,XXY/47,XYY	
Positive	3	1	1	1	1	7	1	1	4	3	7
True-positive	2	1	0	0	1	0	1	0	0	3	2
False-negative	0	0	0	0	0	0	0	0	0	0	0
Specificity	99.92	100	99.92	99.92	100	99.48	100	99.85	99.41	100	99.27
Sensitivity	100	100	100	100	100	100	100	100	100	100	100

### Fetal-free DNA concentration

Among the 2010 samples, the fetal concentration was greater than 4% with no test failure. The mean cff-DNA concentration in the NIPT group was 15.12 ± 6.11%, while the mean cff-DNA concentration in the NIPT-plus group was 12.18 ± 7.03%.

## Discussion

The prevalence of twin pregnancies has increased greatly due to the advanced maternal and paternal age across society as well as the increased use of ART. Among the 42,638 women who underwent NIPT/NIPT-plus during our study period, 2010 women (4.7%) were pregnant with twins. At present, NIPT/NIPT-plus is increasingly widely used in screening singleton pregnancy, with excellent specificity and sensitivity. However, there have been relatively few studies on the performance of NIPT/NIPT-plus for twin pregnancies screening [15, 16]. In this study, we evaluated the specificity and sensitivity of NIPT and NIPT-plus in twin pregnancies to provide a reference value for clinical application.

### Detection efficiency

The screening-positive rates of NIPT and NIPT-plus were 1.65% (22/1331) and 2.95% (20/679), respectively, which were consistent with previous studies showing a 1.9–8.1% screening-positive rate for aneuploidy in twins [5, 17, 18]. Because of the relative rarity of affected twin pregnancies, robust estimates of sensitivity are difficult to establish. In our study, three cases of T21, one case of T18, one case of T7, two cases of SCAs, and seven cases of CNVs were screening-positive by NIPT, which were confirmed as positive in 2, 1, 0, 1, and 0 cases, respectively. One case of T21, one case of T7, seven cases of SCAs, and seven cases of CNVs were screening-positive by NIPT-plus, which were confirmed as positive in 1, 0, 3, and 2 cases, respectively. No false-negative result was reported, and the sensitivities and specificities were above 99% in both the NIPT and NIPT-plus groups, in line with Palomaki et al. [19] and Khalil et al. [20].

In this cohort, all the screening positives of 47,XXY/47,XYY by NIPT/NIPT-plus were confirmed as true positives, while all the screening positives of 45,X were confirmed as false positives, in line with previous reports that NIPT/NIPT-plus performed better in predicting 47,XXY/47,XYY than 45,X [21, 22]. Possible reasons include homologous genes on the X and Y chromosomes, low guanosine cytosine content on the X chromosome, nonrandom inactivation of the X chromosome in placental tissue (mostly the paternal X chromosome, which tends to be inactivated in XX trophoblasts), and age-related X chromosome loss in normal female white blood cells [23].

In one of our previous research, NIPT-plus had a better performance in detecting CNVs than NIPT in singleton pregnancy (the detection rate increased by 1.02%) [24]. But in this study, all CNVs detected by NIPT and 71.43% of CNVs detected by NIPT-plus were false positives. It showed that the effectiveness of NIPT in detecting CNVs is poor, while NIPT-plus has a certain detection efficiency (the positive predictive value (PPV) was 28.57%). The reason may be that the resolution efficiency was closely related to sequencing depth and coverage, sequence matching, repeat sequences, homologous sequences, the complexity of twin pregnancies, and other factors that can lead to false positives [25]. Some studies have shown that increasing the sequencing depth can effectively improve CNVs sensitivity, but the cost is higher [26]. In this area, more research is needed in the future.

One shortcoming of this study is the lack of follow-up of some false-positive cases. One false-positive case of T21 was found in the NIPT group. The case was conceived spontaneously as monochorionic diamniotic (MCDA) with a BMI of 25 and a fetal concentration of 5.35%. The possible reasons for false positives were confined placental mosaicism (CPM), maternal chromosome abnormality, or vanishing twin syndrome [27, 28]. One false-positive case of T7 was detected by NIPT and one by NIPT-plus. The frequency of T7, which was the most common RAT in both NIPT and chorionic villus sampling (CVS) datasets, was comparable, at 0.0746% and 0.0795%, respectively [29]. Most fetuses with T7 are thought to be spontaneously aborted during the first trimester. Therefore, after 12 weeks of gestation, a positive result of NIPT for T7 conventionally indicates mosaic T7 in the placenta and/or in the fetus [30]. Therefore, two cases of T7 false positives were considered to be caused by CPM. The incidence of CPM in typical CVS samples was approximately 1–2% [31]. The principal mechanism of formation of the abnormal mosaic cell line is a nondisjunction error (either meiotic or mitotic), which gives rise to an aneuploid cell line. Thus, most autosomal trisomies remain confined to the placenta, as they are almost universally lethal, either in a homogeneous or in a mosaic form, when affecting the fetus [32]. CPM remains a biologically based limitation of any indirect method of fetal DNA assessment at present.

**Fig. 1:**
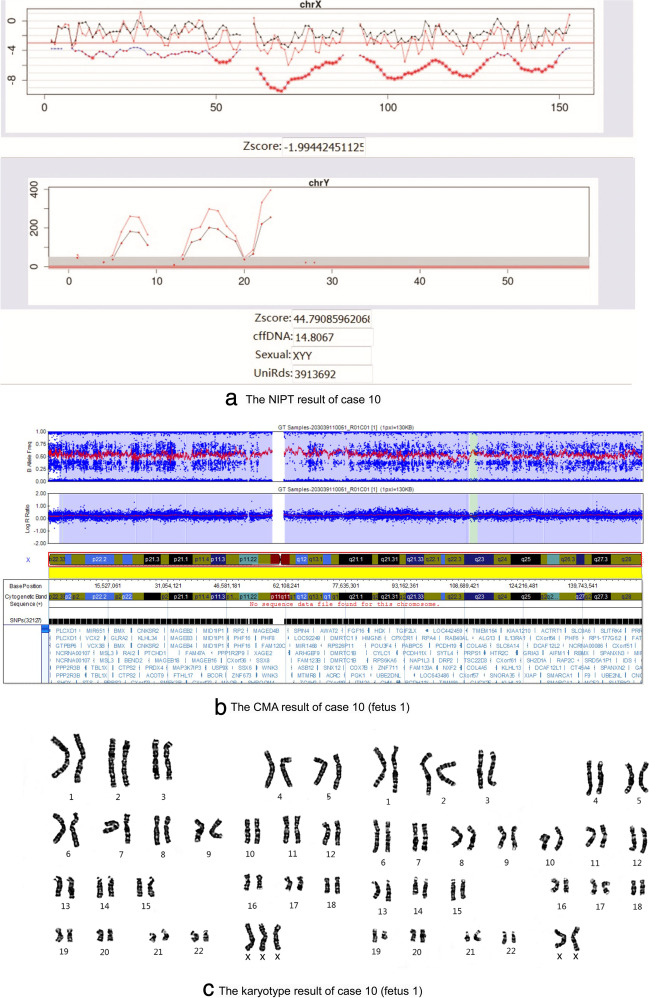
NIPT, CMA, and karyotype results of case 10. **a** The NIPT result of case 10. **b** The CMA result of case 10 (fetus 1): arr[hg19]Xp22.33q28(2,699,676-154,921,994)×2-3. **c** The karyotype result of case 10 (fetus 1): mos47,XXX, 21pss(28)/46, XX, 21pss(22)

The two pregnant women of the 47,XXY fetuses were 40 and 38 years old, respectively, while the pregnant woman with the 47,XYY fetus was 32 years old. The frequencies of 47,XXY and 47,XYY were correlated with maternal age, similar to the results reported by Zhu et al. [33]. It was noteworthy that case 10 was 47,XXY screening-positive by NIPT; the CMA results of her amniotic fluid showed that fetus 1 was arr[hg19]Xp22.33q28(2,699,676-154,921,994)×2-3 and fetus 2 was 47,XY; the karyotype of cord blood showed that fetus 1 was mos47,XXX, 21pss(28)/46,XX, 21pss(22) and fetus 2 was 47,XY. Because one of the twins was a normal male and the other had a mosaicism of 47,XXX, so that the overall fetal free DNA showed more X signal than normal, the NIPT result was 47,XXY. The incidence rate of trisomy X syndrome is approximately 1/1,000 in the female population. The clinical phenotypes vary among individuals. In case 10, a boy and a girl were born prematurely at 32 weeks of gestation due to premature rupture of membranes. No physical or developmental abnormalities were found in these twins during the 6-month follow-up after birth. The NIPT, CMA, and karyotype results of case 10 are shown in Fig. 1.

Cases 6 and 16 in the NIPT-plus group were confirmed to be positive for CNVs. Case 6 had a 3 Mb duplication of chromosome 16. After amniocentesis, the CMA results showed that both fetuses had a 2.9 Mb microduplication at 16p13.11-p12.3. This region overlaps the 16p13.11 microduplication syndrome (DECIPHER database) region. Some studies have shown that it is a benign variation [34], but other studies have shown that it may be related to some neurodevelopmental symptoms (such as intellectual disability and autism) [35]. Both parents then underwent a CMA examination, which found that the abnormality was inherited from the asymptomatic father. After genetic counseling, the couple chose to continue the pregnancy, and two girls were born healthy. The NIPT-plus results of case 16 showed a 6 Mb deletion on chromosome 13. After amniocentesis, the CMA results showed that one of the twins had a 5.9 Mb microdeletion at 13q21.33-q22.1. A fragment involving 25 genes, 13 of which were OMIM genes, and no haploinsufficiency gene was identified. In the DECIPHER database, we found a similar case (400849) with symptoms of language delay, microcephaly, and hypotonia. There is no similar microdeletion in the DGV database, and the existing data cannot determine whether this CNV would be pathogenic. After genetic counseling, the couple chose to continue the pregnancy and had a stillborn girl and a girl born healthy. The cause of stillbirth was unknown. Since no further chromosome examination was performed, it was unknown whether the stillborn fetus was the fetus with the deletion.

### Chorionicity

Ten cases of chromosomal abnormalities were found and confirmed in this study, eight DCDA and two MCDA. The rate of the MCDA abnormality was 0.45% (2/440), and that of the DCDA was 0.54% (8/1,493). In our study, all the DCDAs had only one of the twins affected, while all the MCDAs had both fetuses affected. This confirms the importance of chorionicity in fetal chromosomal abnormalities of twin pregnancies [17]. It is generally believed that MCDA arises from the same fertilized egg and that the genomes of the two fetuses are the same; DCDA twins can be monozygotic or dizygotic, and the genetic materials of the two fetuses can be the same or different. The theoretical risk of aneuploidy in monozygotic twins is similar to that of singleton pregnancy. In dizygotic twins, it is approximately twice as high as in a singleton pregnancy, and the likelihood of both fetuses being affected is low [36]. In addition, some studies have reported discordant karyotypes of MCDA twins [37]. The mechanism may be a gain or loss of mitotic chromosomes before and after the formation of twins. Therefore, samples of the two fetuses should be acquired for prenatal diagnosis to avoid missed diagnosis. Otherwise, because MCDA twins share one chorionic villus, any inconsistency between MCDA twins cannot be detected in the chorionic villi extracted for prenatal diagnosis. Therefore, chorionic villus sampling is not recommended [38].

### Mode of conception

The majority of women included had used ART (54.38%), which was consistent with the findings of Tan et al. [39]. In this study, the rates of chromosome abnormalities in pregnancies conceived spontaneously and in pregnancies conceived by ART were 0.44% (4/917) and 0.55% (6/1093), respectively. The difference was not significant between the two modes of conception, consistent with the results of Yu et al. [40].

### Fetal fraction

According to some reports, the overall fetal fraction is higher in twin pregnancies than in singleton pregnancy, but the individual contribution from each fetus is lower [36, 41]. In our study, the concentration of cff-DNA in the NIPT group (15.12±6.11) was higher than that in the NIPT-plus group (12.18±7.03). The reason may be that the average gestational age of the NIPT group (17.04±4.03) was older than that of the NIPT-plus group (16.21±3.91) (the fetal fraction increases with gestational age), and the number of samples in the NIPT group was greater than that in the NIPT-plus group. Regression analysis confirmed that the number of cases and gestational age of the two groups did affect the fetal concentration (*P* < 0.05). In addition, since the fetal fraction in both groups exceeded the reporting standard (>4%), this difference did not affect the results of this study.

### Clinical indications

Analyzing the clinical indications of the two groups, we found that the top 3 clinical indications of NIPT were voluntary testing, ART, and intermediate risk of serological screening, but those of NIPT-plus were ART, voluntary testing, and intermediate risk of serological screening. We found that people's awareness of NIPT/NIPT-plus in pregnant women has grown, pregnant women who undergo ART are more cautious than those who conceive spontaneously, and pregnant women with previous adverse outcomes of pregnancy or a previous pregnancy history of chromosomal abnormalities in the fetus are more likely to choose NIPT-plus. Among the 10 true-positive cases in this study, abnormal serological screening was the most common indication, accounting for 40% (4/10); ART accounted for 30% (3/10); an abnormal result of first-trimester ultrasound (NT thickening), voluntary testing and previous adverse outcome of pregnancy each were responsible for 10%. Therefore, it is necessary to carry out serological screening and ultrasound examination in the early stage of twin pregnancies; pregnant women with previous adverse outcomes of pregnancy or previous pregnancy history of fetal chromosomal abnormalities and ART should pay more attention to prenatal screening for fetal chromosomal abnormalities [42].

### Pregnancy outcome of true-positive cases

Regarding the pregnancy outcomes of positive cases, we found that two cases of CNVs were inherited from asymptomatic parents, and the two couples chose to continue the pregnancies; in the four cases of common trisomy, the parents chose to reduce the affected fetus because of the chance of severe intellectual disability and other serious abnormalities. Among the SCAs cases, one pregnant woman with 47,XXX fetus, one pregnant woman with 47,XYY fetus, and one pregnant woman with 47,XXY fetus chose to continue the pregnancies, while one pregnant woman with 47,XYY fetuses (both affected) chose to terminate the pregnancy. Seventy-five percent (3/4) of pregnant women with SCAs fetuses chose to continue the pregnancy, in line with So et al. [43]. The reason may be that the clinical phenotype of SCAs is relatively mild [44].

### Pregnancy outcome of screening-positive cases who had no invasive prenatal diagnosis

In the NIPT/NIPT-plus screening-positive cases that had no invasive prenatal diagnosis, two cases had no follow-up information. One case of T18, 1 case of 10 dup30Mb, 1 case of T21, 1 case of 47,XXX and 1 case of T20 mosaicism had adverse pregnancy outcomes, including abortion due to growth retardation or multiple malformations, stillbirth, and heart disease detected after birth. We speculate that these may be related to the chromosomal abnormalities in the fetuses. One case of T7, one case of 8 dup15Mb, one case of 47,XYY, one case of 45,X/46,XY, and one case of 16 del2Mb showed no abnormalities during the follow-up period. This may be related to the high false-positive rate of NIPT/NIPT-plus for these types of chromosomal abnormalities. Moreover, given the special nature of microdeletion/microduplication syndromes and SCAs, these cases need to be followed up for a long time.

The study included a relatively large sample and evaluated the specificities and sensitivities of NIPT and NIPT-plus in twin pregnancies to provide a reference for clinical application in prenatal screening. The shortcomings were false-positive cases that were not verified through the placental testing after delivery, few positive cases, insufficient follow-up time, and few details in the follow-up information. One reason was that some patients were not cooperative with the follow-up, and another was the relative lack of funds.

## Conclusions

Integrating all the information described above, NIPT and NIPT-plus have excellent performance in detecting chromosome aneuploidies in twin pregnancies. But for CNVs, the effectiveness of NIPT is poor, and the NIPT-plus have a certain detection efficiency. Given that there is no better method for screening for chromosomal abnormalities in twins currently, NIPT/NIPT-plus can be the preferred screening method. It is worth noting that pre- and post-test genetic counseling is especially important, and the impact of chorionicity, mode of conception, and fetal fraction should be taken into consideration. Invasive prenatal diagnosis is required for high-risk cases due to the possibility of false-positive.

## Data Availability

The datasets used and/or analyzed during the current study are available from the corresponding author on reasonable request.

## Abbreviations

CNVs: copy number variations
T21: trisomy 21
SCAs: sex chromosome aneuploidies
T18: trisomy 18
T13: trisomy 13
NIPT: noninvasive prenatal testing
cff-DNA: cell-free fetal DNA
NIPT-plus: expanded noninvasive prenatal testing
RATs: rare autosomal trisomies
ACMG: American College of Medical Genetics and Genomics
ART: assisted reproductive technology
FCAPS: fetal copy number analysis through maternal plasma sequencing
CMA: chromosomal microarray analysis
AMA: advanced maternal age
NT: nuchal translucency
β-hCG: β-human chorionic gonadotropin
AFP: alpha-fetoprotein
uE3: unconjugated estriol
DCDA: dichorionic diamniotic
T7: trisomy 7
MCDA: monochorionic diamniotic
CPM: confined placental mosaicism
CVS: chorionic villus sampling
MCMA: monochorionic monoamniotic
SC: spontaneously conceived
PPV: positive predictive value
